# Supplementary material for: Towards understanding the antagonistic activity of phytic acid against common foodborne bacterial pathogens using a general linear model
Source: PLoS One. 2020 Apr 17;15(4):e0231397. doi: 10.1371/journal.pone.0231397 (PMC7164649; doi:10.1371/journal.pone.0231397)
Supplement: S1 Data — (PDF) [file pone.0231397.s001.pdf]

MIC phytic acid against indicator strains

Indicator strains / MIC (mg/ml)	*L. monocyt*	*S. aureus*	*S.* Typhi	*P. aeruginos*	*E. coli*
phytic acid	0.97	0.488	0.244	0.244	0.244
citric acid	2.5	1.25	1.25	1.25	1.25
standard deviation	0.06	0.054	0.041	0.052	0.064

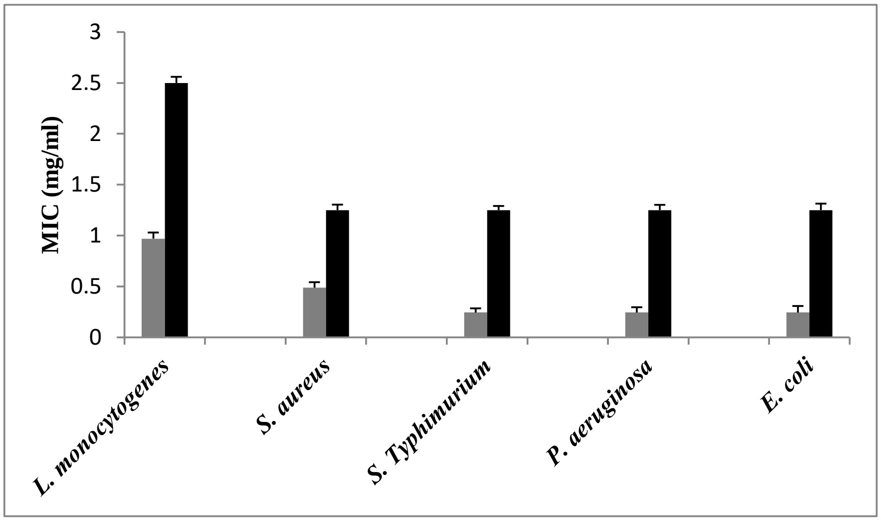

inhibitory spectrum (mm) of phytic acid against *L monocytogenes* , *S aureus* and *S* Typhimurium

	Phytic acid concentratio					
	1.22	2.44	4.88	7.32	9.76	12.2
L. monocytogenes	0.00±0.00[a]	0.00±0.00[a]	0.00±0.00[a]	0.00±0.00[a]	8.00±1.00[a]	11.50±0.25[a]
S. aureus	0.00±0.00[a]	14.75±0.75[c]	17.75±1.25[c]	20.75±1.25[c]	22.75±1.50[c]	24.25±0.75[c]
***S.* Typhimirium**	8.25±0.25[b]	11.00±0.25[b]	15.25±1.00[b]	17.25±1.50[b]	20.00±1.00[b]	21.00±0.75[b]

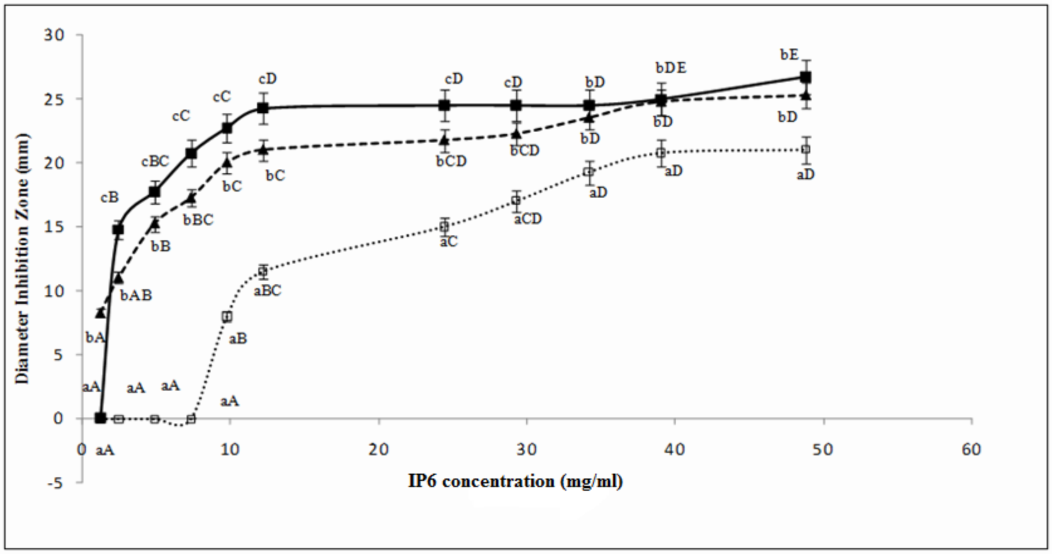

Influence of the dose of IP6 on the growth of *L. monocytogenes* ATCC 19117

	Control	1 MIC	2 MIC	4 MIC	5 MIC
0	3.305351	3.3053514	3.3053514	3.3053514	3.30535137
3	6.089905	6.0899051	6.0899051	6.0899051	6.08990511
4	6.491362	5.69897	5.5563025	4.9242793	4.30103
5	7	5.748188	4.5314789	1	1
6	8	5.7781513	4.0170333	1	1
8	9	5.2	3.5	1	1
12	12	4.8	3.02	1	1
16	12.5	4.3	2.43	1	1
20	12.8	3.9	1.8	1	1
24	13	3.5910646	1	1	1

	**Control**	**1 MIC**	**2 MIC**	**4 MIC**	**5 MIC**
**0**	3.305Aa	3.305aA	3.305aC	3.305aB	3.305aB
**3**	6.089aB	6.089aC	6.089aF	6.089aD	6.089aD
**4**	6.491dB	5.698cBC	5.556cE	4.924bC	4.301aC
**5**	7dCD	5.748cBC	4.531bDE	1aA	1aA
**6**	8Dd	5.778cBC	4.017bD	1aA	1aA
**8**	9Dd	5.2cB	3.5bC	1aA	1aA
**12**	12dE	4.8cAB	3.02bBC	1aA	1aA
**16**	12.5dEF	4.3cAB	2.43bB	1aA	1aA
**20**	12.8dEF	3.9AcA	1.8bAB	1aA	1aA
**24**	13cF	3.59bA	1aA	1aA	1aA

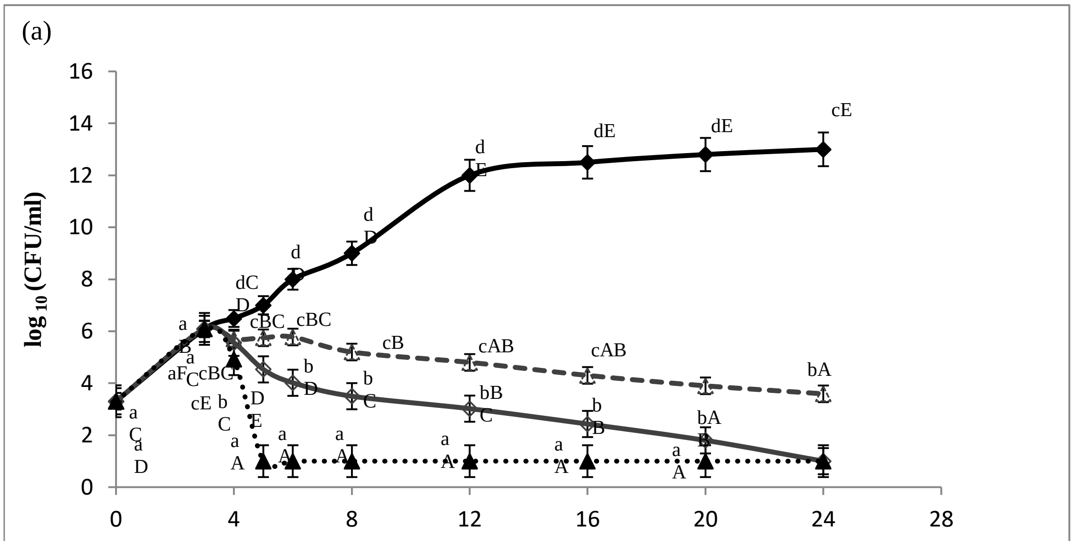

Incubation time (h)

Influence of the dose of IP6 on the growyh of *S. aureus* ATCC 6538

	Control	1 MIC	2 MIC	4 MIC	5 MIC
0	4.060698	4.0606978	4.0606978	4.0606978	4.06069784
3	6.374748	6.3747483	6.3747483	6.3747483	6.37474835
4	7.10721	5.90309	5.6532125	2.8129134	2.53
5	8.041393	5.9242793	5.0170333	1	1
6	8.681241	5.9542425	3.3138672	1	1
8	8.87	5.2	3	1	1
12	9.2	4.6	2.4	1	1
16	9.42	4	2	1	1
20	9.6	3.7	1.5	1	1
24	9.681241	3.3159703	1	1	1

	**Control**	**1 MIC**	**2 MIC**	**4 MIC**	**5 MIC**
**0**	4.06aA	4.06aAB	4.06aCD	4.06aC	4.06aC
**3**	6.374aB	6.374aD	6.374aE	6.374aD	6.374aD
**4**	7.107cBC	5.903bC	5.653bD	2.812aB	2.53aB
**5**	8.041cBC	5.924bC	5.017bD	1aA	1aA
**6**	8.681dBC	5.954cC	3.313bC	1aA	1aA
**8**	8.87dBC	5.2cBC	3bC	1aA	1aA
**12**	9.2dC	4.6cB	2.4bBC	1Aa	1aA
**16**	9.42dC	4cAB	2bB	1aA	1aA
**20**	9.6dC	3.7cA	1.5bAB	1aA	1aA
**24**	9.681cC	3.315bA	1aA	1aA	1aA

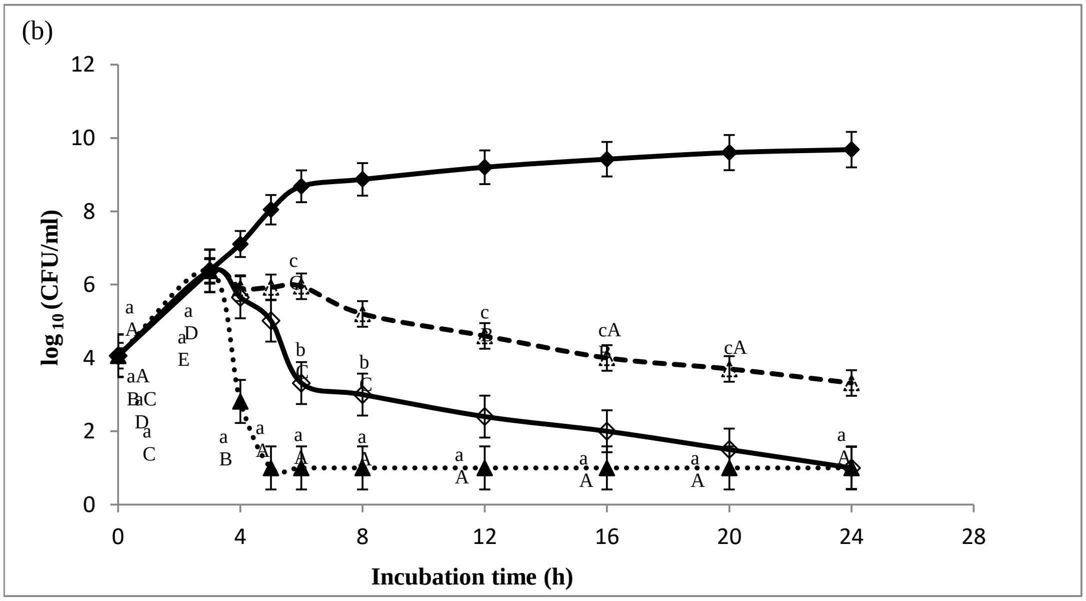

Influence of the dose of IP6 on the growth of S. Typhimurium ATCC 14028

	Control	1 MIC	2 MIC	4 MIC
0	4.068186	4.0681859	4.0681859	4.0681859
3	5.880814	5.8808136	5.8808136	5.8808136
3.1	5.9274	4.35	4.21	3.2
3.3	5.947	3.57	3.48	2.7
3.5	5.951	2.8	2.68	1.97
3.6	5.9576	2.041	1.987	1.32
3.8	5.967	1.32	1.23	1.14
4	5.9741	1	1	1
5	5.987	1	1	1
6	6.008	1	1	1
8	6.225	1	1	1
12	6.5	1	1	1
16	6.6	1	1	1
20	6.75	1	1	1
24	6.9	1	1	1

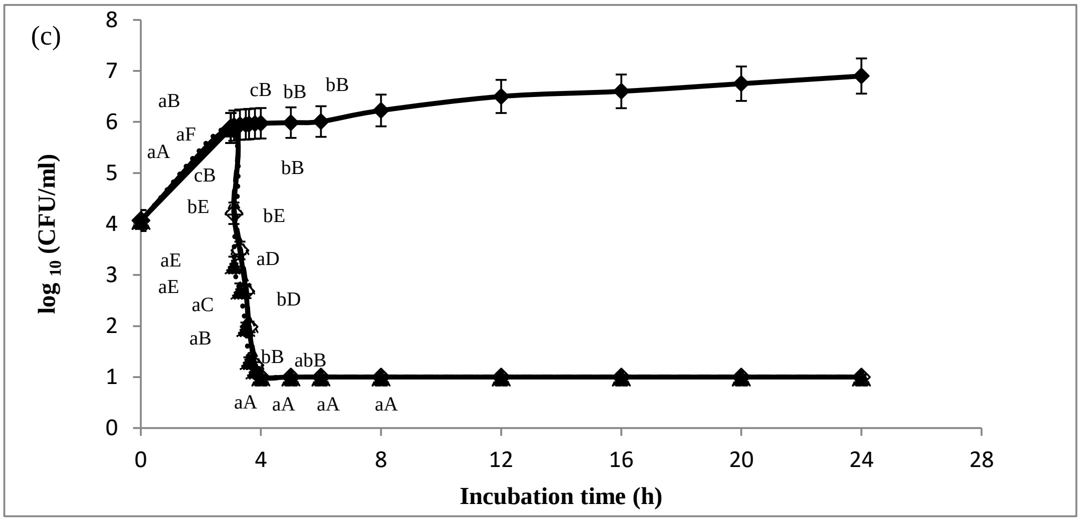

n (mg/ml)				
24.4	29.28	34.16	39.04	48.8
15.00±1.25[a]	17.00±1.50[a]	19.25±1.25[a]	20.75±2.00[a]	21.00±1.75[a]
24.50±1.50[c]	24.50±1.75[c]	24.50±1.00[b]	25.00±1.25[b]	26.75±2.00[b]
21.75±1.50[b]	22.25±1.50[b]	23.50±1.75[b]	24.75±1.75[b]	25.25±1.75[b]
